# Analysis of somatostatin receptors and somatostatin promoter methylation in human gastric cancer

**DOI:** 10.3892/ol.2013.1614

**Published:** 2013-10-10

**Authors:** XUEFEI SHI, XIAO LI, LIN CHEN, CHUNHUI WANG

**Affiliations:** Department of Gastroenterology, West China Hospital, Sichuan University, Chengdu, Sichuan 610041, P.R. China

**Keywords:** somatostatin, receptor, methylation, gastric cancer

## Abstract

Somatostatin (SST) is a gut peptide that is able to inhibit the growth of tumor cells in gastric cancer and other types of cancer. The present study investigated the mRNA and protein levels of SST and SST receptors (SSTRs) in human gastric cancer, and detected the DNA methylation of the SST promoter. The protein levels of SST were detected using a radioimmunoassay in 102 human gastric tissue specimens (51 pairs of samples from 51 gastric cancer patients, each pair of samples included a cancer tissue and a normal tissue sample). SST and SSTR mRNA expression was assessed by reverse transcription-PCR (RT-PCR), while SST promoter methylation was examined using quantitative methylation-specific PCR (qMSP) in 51 pairs of tissues. The association between SST protein and RNA levels and SST methylation and gastric cancer were also analyzed. The protein levels of SST were decreased in the gastric cancer group compared with those of the normal group (5.091±0.994 vs. 7.399±0.956 pg/mg; P<0.01). The RT-PCR analysis indicated that the mRNA levels of SST (0.218±0.183 vs. 0.456±0.331; P<0.001) and SSTRs in the gastric cancer group were lower compared with those of the normal gastric tissue group. The methylation proportion of SST was 45.1% (23/51) in the carcinoma group and 3.9% (2/51) in the normal group. In conclusion, SST promoter methylation is a common event in human gastric cancer and is connected with a decrease in SST protein and RNA levels and associated with gastric carcinogens.

## Introduction

Gastric cancer is the third most common type of cancer worldwide, with high incidence and mortality; ~989,600 new cases of gastric cancer are diagnosed, of which 738,000 cases succumb, per year, according to GLOBOCAN 2008 (1). Gastric cancer has developed into a serious health problem worldwide, particularly in Eastern Asia, Eastern Europe and South America. The transcriptional inactivation of tumor suppressor genes is one of the main reasons for carcinogenesis. Epigenetics studies have confirmed that DNA methylation in the promoter region of a tumor suppressor gene leads to transcriptional inactivation and is correlated with the carcinogenesis of gastric cancer (2–5). This phenomenon has also been simultaneously discovered in gastric cancer tissue. Somatostatin (SST) is a gut peptide that is able to inhibit the growth of tumor cells in gastric cancer and other types of cancer, and is regarded as a new cancer repressive polypeptide (6–9) However, the further mechanistic interaction between gastric tumorigenesis and SST promoter methylation remains unclear.

The present study investigated the expression of SST, SST mRNA and SSTR mRNA in gastric cancer and utilized methylation-specific PCR (MSP) technology for the analysis of SST promoter DNA methylation.

## Materials and methods

### Tissues samples

A total of 51 pairs of fresh gastric tissue samples were obtained from the Department of General Surgery, West China Hospital (Sichuan University, Chengdu, China). Each pair of samples, which consisted of a gastric cancer tissue and a normal gastric tissue sample, was divided into a gastric cancer group and a normal gastric tissue group, respectively. All the tumor and normal gastric mucosal epithelial tissues were histologically verified. The patients were not administered radiation, chemical or biological treatment prior to surgery. Written informed consent was obtained from each patient before enrollment and this study was approved by the Ethics Committee of West China Hospital.

### Radioimmunoassay analysis of SST

Following the homogenization of the normal and gastric cancer tissues, the total protein level was treated with the Total Protein Reagent kit (Biosino Bio-technology and Science Inc., Hong Kong, China) at 37°C for 10 min and measured at a wavelength of 546 nm using an allophanamide assay. The homogenate was then treated using an SST radioimmunoassay kit (HY-104; Beijing Sino-UK Institute of Biological Technology, Beijing, China). The protein level of SST was measured using a γ-911 radioimmunoassay counter (Zhongjia Optical and Electrical Instrument Company, Hefei, China). The level of SST was corrected using the total protein level.

### Detection of SST and SSTR mRNA levels using RT-PCR

Total RNA was isolated by Trizol (Invitrogen, Carlsbad, CA, USA). First-strand cDNA was produced using the Reverted Aid First Strand cDNA Synthesis kit (Fermentas, Pittsburgh, PA, USA). The primer sequences and reaction conditions for SST and SSTRs are listed in Table I.

### Detection of SST DNA methylation using quantitative MSP (qMSP)

Genomic DNA was isolated using the Tissue Gen DNA kit (ComWin Biotech, Beijing, China). Subsequently, the positive methylated controlled DNA that was extracted from the placenta tissue was incubated with CpG methyltransferase for 1–2 h at 37°C and terminated following an incubation period at 65°C for 20 min. Following this, all the genomic DNA, including the positive methylated control (placenta tissue DNA) were managed by DNA methylation modification using the Methylamp DNA Modification kit (ComWin Biotech) and incubated for 60 min at 80°C in the dark. The tissue DNA treated with CpG methyltransferase was defined as the positive methylation control and the double distilled water as negative. The determination of SST DNA methylation for all the modified genomic DNA was performed using qMSP. The primer sequences, reaction conditions and cycles for SST are listed in Table I. The objective products of qMSP were verified by a sequence assay.

### Statistical analysis

SPSS 16.0 statistical software (SPSS Inc., Chicago, IL, USA) was used for the statistical analysis. The measurement data was analyzed using a paired-samples t-test and presented as the mean ± standard deviation. P<0.05 was considered to indicated a statistically significant difference. The count data were applied using the χ^2^ and Fisher’s exact tests (P<0.05).

## Results

### SST protein levels in gastric cancer

**Figure 1:**
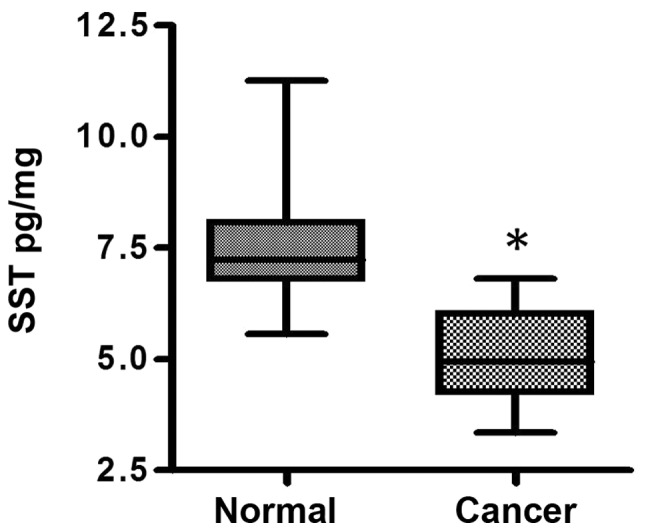
Expression of SST protein in 51 cancer samples and their corresponding normal samples from the same patients. Total protein level was isolated from the homogenized tissues and measured. An SST radioimmunoassay kit was used to analyze SST in the gastric cancer and normal gastric tissues. ^*^P<0.01 for the cancer group vs. the normal group. SST, somatostatin.

The SST protein level in the normal group was significantly higher compared with that in the cancer group (7.399±0.956 vs. 5.091±0.994 pg/mg; P<0.01; Fig. 1).

### Detection of SST, SSTR2, SSTR3 and SSTR5 mRNA in the normal and cancer groups using RT-PCR

**Figure 2:**
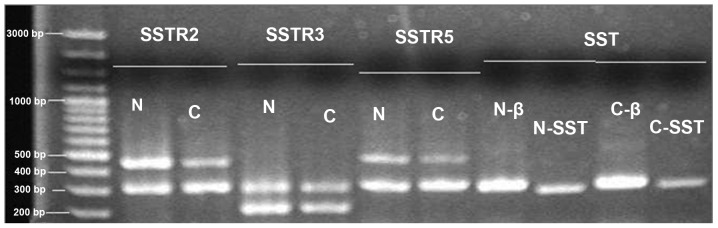
Expression of mRNA for SST, SSTR2, SSTR3, SSTR5 in normal and cancer groups. Total RNA was isolated from normal gastric tissue and gastric cancer. RT-PCR was used to amplify SST and SSTR cDNAs with gene-specific primers. PCR products were separated on 1.5% agarose gel and visualized following ethidium bromide staining. N, normal group; C, cancer group; β, β-actin; SST, somatostatin, SSTR, SST receptor; RT-PCR, reverse transcription-PCR.

**Figure 3:**
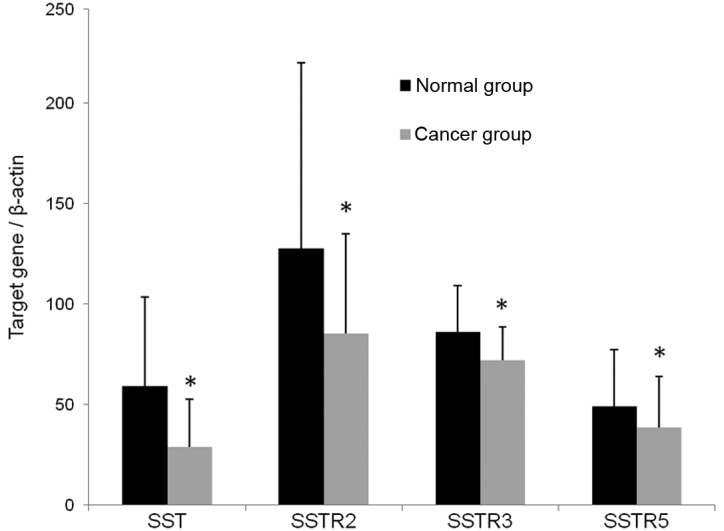
Comparison of SST and SSTR mRNA expression in the normal and cancer groups (n=51). Results are the mean ± SE from three experiments. ^*^P<0.01 for the cancer group vs. the normal group. SST, somatostatin; SSTR, SST receptor.

The expression of SST, SSTR2, SSTR3 and SSTR5 mRNA in the normal and cancer groups was detected using RT-PCR. The lengths of the amplified products of SST, SSTR subtypes and β-actin are marked (Fig. 2). The mRNA levels were analyzed using the integral optical density (IOD) by the Quantity One software (Bio-Rad, Hercules, CA, USA). The IOD of β-actin was used as the standard to correct the IOD levels of SST, SSTR2, SSTR3 and SSTR5. The value indicated that the SST and SSTR mRNA expression levels in the cancer group were lower than those of the normal group. The IOD ratio of SST in the two groups was 0.456±0.331 vs. 0.218±0.183 (P<0.001). The IOD ratios of SSTR2, SSTR3 and SSTR5 in two groups were (0.900±0.396, 0.647±0.174 and 0.364±0.202 vs. 0.646±0.375, 0.538±0.125 and 0.299±0.188, respectively (P<0.01; Fig. 3).

### DNA methylation of the SST gene correlates with SST expression

The DNA methylation level of SST in the two groups was detected using qMSP. A single melt peak demonstrated that the quality of the primer was high and no useless introductions were identified in the assay. When the reaction reached the concentration threshold (CT), the CT value was 10–13 reaction cycles in the amplification curves, which demonstrated that the amplification was effective (Table II).

**Figure 4:**
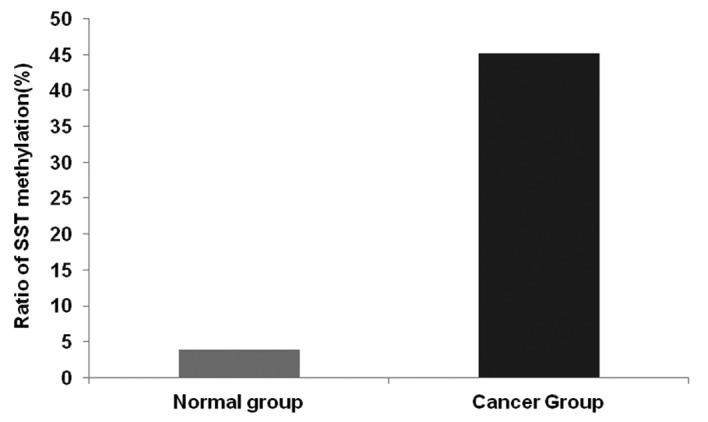
Comparison of the ratio of DNA methylation in the two groups. Genomic DNA was isolated using the Tissue Gen DNA kit. The determination of SST DNA methylation for all the modified genomic DNA was performed using qMSP. The objective products of the qMSP were verified using a sequence assay. SST promoter DNA methylation was identified in 23 of 51 cancer samples and two of 51 normal samples. SST, somatostatin; qMSP, quantitative methylation-specific PCR.

**Figure 5:**
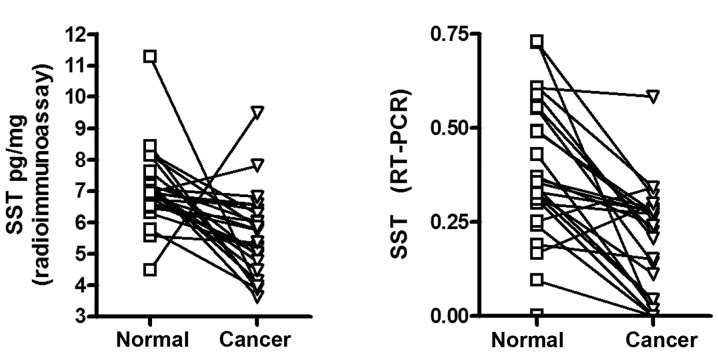
Correlation between DNA methylation and SST levels. Protein and mRNA levels of SST in 25 methylation patients were analyzed side by side for comparison, revealing that DNA methylation of the SST gene correlated with silencing of (A) protein and (B) mRNA expression. SST, somatostatin.

The positive rate of DNA methylation for SST in the carcinoma group was markedly higher than that of the normal group (23/51 vs. 2/51; Fig. 4). The distribution of the positive methylation cases for SST in patients with gastric cancer did not correlate with the age or gender of the patients or the size, location and type of the carcinoma. In the 25 patients with DNA methylation of SST, the protein and mRNA levels of SST were significantly lower in the non-methylation tissues (Fig. 5).

## Discussion

The occurrence and development of gastric cancer has been regarded as a long-term, multi-phase and multi-gene co-impacting process that is affected by various external factors and genetic mutations, which manifest as gastrointestinal diseases and external disorders (10). Among them, the lack of genetic fragmentation, mutation and the methylation of the DNA promoter region are considered as main factors that lead to the emergence and development of human cancer at a molecular level. A number of studies have suggested that numerous tumor suppressor genes undergo methylation of the DNA promoter region in gastric cancer, including p16, hMLH1, Runx3, PTEN and XAF1 (11–15). The genes are involved in gene repair, cell signal transduction, apoptosis, cell cycle regulation and angiogenesis. Sections of the gene that are rich in CpG dinucleotides are called the CpG islands, which are significant targets for DNA methylation. CpG islands exist in >60% of gene promoter regions, but are not evenly distributed within the genome (16,17). CpG islands are often located in the regulatory area of eukaryotic housekeeping genes, and the potential inactivating effect of methylation has gained interest with regard to the association between the DNA methylation of the CpG islands in tumor suppressor gene promoter regions and cancer.

SST is widely distributed in the gastrointestinal tract and plays a significant role in maintaining the internal environment homeostasis. Several *in vitro* and *in vivo* studies have suggested that SST functions as a tumor suppressor gene in human cancers and may inhibit tumor growth through mechanisms that involve the inhibition of growth factors and hormones and a reduction in the vascularization (6,7,18). The present study identified that the protein level of SST in the normal group was 7.399±0.956 pg/mg, which was significantly higher than that in the cancer group (5.091±0.994 pg/mg). Furthermore, the current study demonstrated that SST mRNA expression was significantly lower in the tumor group (0.218±0.183) compared with that in the normal group (0.456±0.331), as measured using RT-PCR. However, the role of DNA methylation in decreasing the expression of SST in gastric cancer remains undetermined.

The present study used qMSP technology for the analysis of promoter DNA methylation in SST. An increased DNA methylation level was detected in the gastric cancer tissues compared with that in the normal gastric mucosa samples. This result suggested that epigenetic mechanisms may be the leading cause of silencing SST expression in gastric cancer. In addition to gastric cancer (9), DNA hypermethylation has also been identified in colon and esophageal cancer (19,20). The silencing of SST may be a critical step in gastrointestinal tract carcinogenesis. Several studies have confirmed that SST and its analogs may be able to inhibit the growth of cancers (8,18). Our previous studies also identified that octreotide, as one of the SST analogs, was able to inhibit the growth of gastric cancer and induce the apoptosis of gastric cancer cells *in vitro* and *in vivo*(6,7). Further studies are required to investigate the inhibition of endogenous SST expression in tumor tissues, in addition to exogenous SST expression. As a reversible DNA modification, methylation may be reversed using demethylation drugs, including 5-Aza-dC. Treatment with the demethylation drug reverses the status of methylation and recovers SST mRNA expression. This may contribute to an enhanced curative effect in gastric cancer. At present, this has been confirmed by certain *in vitro* experiments (9,19,20). However, further studies are required in order to apply it to clinical treatment.

SST may predominantly function by directly combining with specific SSTRs 1–5 and subsequently activating a variety of signal transduction pathways. Among the receptors, SSTR2, SSTR3 and SSTR5 are closely associated with gastrointestinal cancer. Therefore, SSTR expression levels and the anti-proliferation effects of SST are closely associated. In the present study, by detecting the mRNA expression levels of SSTRs in the normal and cancer groups using RT-PCR, the SSTR mRNA expression levels in the cancer group were lower than those in the normal group, which revealed that the reduction of mRNA expression occurred for not only SST but also SSTRs. Whether the reduction of mRNA expression for SSTRs was associated with the DNA methylation in the promoter region or the other factors remains to be elucidated. Previous studies have confirmed that the DNA methylation of SSTRs exists in numerous types of cancer cells (21,22).

In summary, the present study demonstrated that SST promoter methylation is frequently observed in gastric cancer tissue and is associated with a decrease in SST protein expression. This observation provides a foundation for targeting SST in the treatment of gastric cancer. However, further studies are required to confirm whether SSTR promoter methylation exists in gastric cancer and how it may be demethylated effectively.

## Figures and Tables

**Table I:** Primer sequences, reaction conditions and cycles.

		Reaction conditions	
		
Primer sequences 5′-3′	Length (bp)	Annealing temperature (°C)	Reaction cycles
RT-PCR			
SST			
Sense: GGC TGC GCT GTC CAT CGT C	285	58.0	36
Antisense: CAG CCA GCT TTG CGT TCT CG			
SSTR2			
Sense: GGT GAA GTC CTC TGG AAT CC	461	63.0	36
Antisense: CCA TTG CCA GTA GAC AGA GC			
SSTR3			
Sense: TCA TCT GCC TCT GCT ACC TG	221	65.0	36
Antisense: GAG CCC AAA GAA GGC AGG CT			
SSTR5			
Sense: GTG CAG GAG GGC GGT ACC	474	62.0	36
Antisense: TGG ACG CGG CTC CGT GGC			
β-actin			
Sense: GAC TAC CTC ATG AAG ATC CT	312	53.0	35
Antisense: GCG GAT GTC CAC GTC ACA CT			
qMSP			
SST			
Sense: GGG GCG TTT TTT AGT TTG ACG T	102	58.2	40
Antisense: AAC AAC GAT AAC TCC GAA CCT CG			

SST, somatostatin; SSTR, SST receptor; RT-PCR, reverse transcription-PCR; qMSP, quantitative methylation-specific PCR.

**Table II:** Distribution of methylation cases for SST in pair of groups.

	Normal group		
		
Cancer group	+	−	Total
+	0	23	23
−	2	26	28
Total	2	49	51

SST, somatostatin.
